# Electroacupuncture Neural Stimulation Mitigates Bladder Dysfunction and Mechanical Allodynia in Cyclophosphamide-Induced Cystitis through Downregulation of the BDNF–TrkB Signaling Pathway

**DOI:** 10.1523/ENEURO.0329-24.2025

**Published:** 2025-03-12

**Authors:** Ying Su, Fei Yang, Jun-Cong Xie, Chi Zhang, Rui-Xiang Luo, Wen-Shuang Li, Bo-Long Liu, Min-Zhi Su

**Affiliations:** Department of Rehabilitation, Department of Urology, The Third Affiliated Hospital, Sun Yat-sen University, Guangzhou 510630, China

**Keywords:** BDNF, bladder pain syndrome, cystitis, electroacupuncture, neuroinflammation, TrkB

## Abstract

Central sensitization plays a critical role in bladder pain syndrome/interstitial cystitis (BPS/IC). Electroacupuncture (EA) nerve stimulation therapy has been broadly acknowledged as an effective means of alleviating chronic pathological pain. However, it remains to be explored whether EA is effective in mitigating pain-sensitive symptoms of BPS/IC and the mechanisms involved. This study aims to investigate the analgesic effect and mechanism of EA therapy. We employed several techniques: mechanical pain threshold tests to assess pain sensitivity, urodynamic studies to evaluate bladder function, western blotting for protein analysis, immunofluorescence for visualizing, and transcriptomics. A rat cystitis model was established through a systemic intraperitoneal injection with cyclophosphamide (CYP). EA therapy was executed by stimulating the deep part of the hypochondriac point. EA treatment was observed to effectively reduce mechanical allodynia, enhance urinary function, suppress the activation of microglial cells, and alleviate neuroinflammation. Additionally, EA demonstrated the capability to downregulate brain-derived neurotrophic factor (BDNF)–tyrosine kinase receptor B (TrkB) signal transduction in the spinal dorsal horn. Transcriptome sequencing has indicated that EA therapy potentially inhibits excitatory neural transmission and modulates several pathways related to longevity. Furthermore, EA therapy has shown efficacy in treating conditions such as Huntington's disease, amyotrophic lateral sclerosis, and prion diseases. In conclusion, by regulating the BDNF–TrkB signaling, EA nerve stimulation can effectively alleviate bladder dysfunction and mechanical allodynia in the CYP-induced cystitis model. Our research elucidates the underlying mechanisms of EA therapy in treating bladder dysfunction and offers new theoretical insights for addressing painful sensitization in BPS.

## Significance Statement

Central sensitization is a major factor in bladder pain syndrome/interstitial cystitis (BPS/IC), making effective pain management crucial. This study explores the potential of electroacupuncture (EA) as a therapeutic approach to alleviate pain and improve bladder function in a rat model of BPS/IC induced by cyclophosphamide. Our findings demonstrate that EA therapy significantly reduces mechanical allodynia, enhances urinary function, and decreases neuroinflammation by modulating brain-derived neurotrophic factor–tyrosine kinase receptor B signaling in the spinal dorsal horn. The research highlights EA's capability to inhibit excitatory neural transmission and provide relief in chronic pain conditions. These results offer new insights into the mechanisms of EA therapy, potentially improving treatment strategies for BPS/IC and similar pain syndromes.

## Introduction

Bladder pain syndrome (BPS), also known as interstitial cystitis (IC), is often referred to as the “social cancer” due to its persistent and difficult-to-treat bladder or perineal pain, accompanied by frequent and urgent urination (Bresler et al., 2019). Many patients endure chronic physical and mental pain, leading to feelings of depression and even suicidal thoughts (Clemens et al., 2019). The underlying pathogenic mechanism of BPS remains elusive, making the current treatments largely ineffective and presenting a significant challenge in urology (Li et al., 2022). There is an observed increase in the prevalence of BPS year over year, currently affecting ∼2.7–6.5% of women (Li et al., 2022). This condition incurs medical expenses that are more than double compared with those without BPS (Li et al., 2022). Therefore, there is a pressing need to explore new therapeutic approaches and delve deeper into the molecular mechanisms associated with BPS.

The pain associated with BPS is linked to increased sensitivity of bladder sensory mechanisms. Emerging research suggests that a central neuroinflammatory response might be a key driver of BPS (Greig et al., 2023). In patients with BPS, dysfunction in the bladder epithelium allows urinary solutes to infiltrate, leading to the activation of mast cells in the mucosal layer and subsequent release of inflammatory mediators (Padilla-Fernández et al., 2022). These factors further stimulate peripheral nerves, including C class nerve fibers and those transmitting nociception (Padilla-Fernández et al., 2022). The activation of these nerves involves several key proteins, notably brain-derived neurotrophic factor (BDNF), neuregulin-1, and NOD-like receptor thermal protein domain-associated protein 3 (Padilla-Fernández et al., 2022; Zhang et al., 2022). BDNF, a critical neurotransmitter in the central nervous system, is produced by neuronal cells and conveyed to nerve terminals through axonal transport (Ding et al., 2020). It is essential for neuronal survival, growth, development, and synaptic plasticity in both the central and peripheral nervous systems (Ding et al., 2020). BDNF exerts its biological functions through its specific binding to the tyrosine kinase receptor B (TrkB; Jhang et al., 2023). While, initially, studies primarily focused on BDNF's impact on motor functions and memory formation, recent research underscores its substantial influence within the central nervous system (Kumar et al., 2023; Zosen et al., 2023). Elevated levels of BDNF have been detected in various animal models of pathological pain, indicating its involvement in pain perception regulation across both central and peripheral nervous systems (Kumar et al., 2023; Zosen et al., 2023). In our prior research, an increase in BDNF expression was observed in the spinal cord's dorsal horn in a BPS model (Ding et al., 2020). Notably, administering a specific TrkB inhibitor, targeting the BDNF receptor, effectively reduced glial cell activation and the release of inflammatory factors, consequently diminishing pain sensitivity in BPS. BDNF promotes the activation of astrocytes and microglia, intensifying neuroinflammation. Additionally, it leads to abnormal bladder sensitivity to mechanical stimuli through the BDNF–TrkB–p38 signaling pathway, resulting in enhanced pain sensation. These results highlight the BDNF–TrkB pathway's potential role in managing BPS pain, offering a promising avenue for developing new treatment strategies for BPS pain.

Electroacupuncture (EA) nerve stimulation therapy is widely acknowledged as an effective method for alleviating chronic pathological pain (Chang et al., 2023; Wan et al., 2023). EA is increasingly recognized as an effective treatment in neurological disorders such as Huntington's disease and amyotrophic lateral sclerosis (Hsu et al., 2020; Liu et al., 2024). Typically, neural signals from deep receptors and nerve endings are relayed to the central nervous system (Cao et al., 2022). However, inflammatory injuries can induce ectopic spontaneous discharges in both the axonal region and the cell body. This irregular electrical activity travels continuously to the spinal cord and creates ripples in the dorsal horn, resulting in heightened sensitivity of the injured receptor neurons, a condition known as nociceptive hypersensitivity (Cao et al., 2022; Vanuytsel et al., 2023). Research indicates that EA can suppress the expression of excitatory amino acids and their *N*-methyl-d-aspartate receptors in the spinal cord, thus diminishing central sensitization and relieving pain hypersensitivity (Gong et al., 2022). Furthermore, EA therapy can inhibit long-range enhancement of synaptic transmission in dorsal horn neurons, further suppressing central sensitization (Weng et al., 2023). Notably, EA activates opioid receptors and inhibits sodium channel activity, reducing spontaneous discharges and thereby alleviating neuropathic pain (Chang et al., 2023; Wan et al., 2023). The pain-relieving effects of EA therapy on chronic pain, including neuralgia, inflammatory pain, and persistent cancer pain, have been demonstrated in numerous clinical and animal studies (Fan et al., 2020; Chang et al., 2023; Wan et al., 2023; Weng et al., 2023). Nonetheless, the effectiveness of EA in addressing pain sensitivity in cystitis and the underlying mechanisms warrant further investigation.

In conclusion, we propose the hypothesis that EA therapy could modulate BPS pain sensitivity by regulating the BDNF–TrkB pathway by diminishing the release of inflammatory factors. This study endeavors to elucidate the influence of EA therapy on neural transmission mechanisms, potentially broadening the scope of EA's clinical applications.

## Materials and Methods

### Animals

Adult Sprague Dawley (SD) rats, each weighing between 250 and 300 g and certified as specific pathogen-free, were obtained from the Laboratory Animal Research Center at Sun Yat-sen University. These rats were randomly allocated into different groups and maintained in separately licensed animal housing units, with a controlled environment of 24°C and a 12 h light/dark cycle. Rats had continuous access to food and water *ad libitum*. All procedures involving these animals were carried out in strict compliance with the ethical welfare guidelines established by the Institutional Animal Care and Use Committee at the Sun Yat-sen University. The ethical application for this study was approved under Grant SYSU-IACUC-2022-001682.

### BPS model construction

Utilizing the protocol we have previously established (Ding et al., 2020), we constructed a BPS rat model using female SD rats. These rats were screened based on their baseline mechanical pain thresholds in the bladder area, ensuring these were within normal limits. Cyclophosphamide (CYP) was administered intraperitoneally at a dosage of 75 mg/kg, with injections repeated every 3 d for a total of three administrations. Commencing with the initial intraperitoneal injection, the mechanical pain threshold in the bladder area was assessed every 3 d to monitor the success of the model development. For exogenous BDNF administration, recombinant protein of BDNF (248-BD-025, R&D Systems) was injected intrathecally at a dose of 3 ng/rat 2 d after every CYP injection.

### EA nerve stimulation therapy

EA therapy was applied to the deep region of the “Ci Liao” point, which corresponds to the area encompassing the second to fourth sacral nerves. The experimental procedure was executed as follows: Firstly, rats were mildly anesthetized with a 1.5% sodium pentobarbital solution, administered intraperitoneally. Secondly, in a quiet environment, each rat's torso was secured onto a wooden frame, allowing the head and limbs to move *ad libitum*. After a resting period of 20 min, EA stimulation was administered. The EA needle was inserted directly into the hypochondriac point, ∼0.5 cm adjacent to the second and third segments of the sacral spinal cord. The needle was inserted to a depth of 30–40 mm and connected to the EA instrument for continuous wave output. The stimulation frequency was set at 20 Hz, and the EA stimulation lasted for 30 min.

For the sham EA (SEA) method, firstly, rats were mildly anesthetized with a 1.5% sodium pentobarbital solution, administered intraperitoneally. Secondly, in a quiet environment, each rat's torso was secured onto a wooden frame, with their head and limbs left free to move. After a resting period of 20 min, SEA stimulation was given. The EA needle was inserted directly into the hypochondriac point, ∼0.5 cm adjacent to the second and third segments of the sacral spinal cord. The needle was inserted to a depth of 30–40 mm and connected to the EA instrument for continuous wave output but was maintained for 30 min without electric stimulation. Behavioral tests for pain in the bladder area followed these procedures.

### Urodynamics of BPS animal model and test for mechanical withdrawal threshold

Following mild anesthesia with a 1.5% sodium pentobarbital solution via intraperitoneal injection, the rats were placed in a supine position with limbs securely fixed. A fully sterilized and lubricated catheter, ∼1 mm in diameter, was then inserted into the bladder. Once the catheter was fully inserted into the urethra and reached the bladder wall, it was secured by retracting it outward by ∼5 mm. Any existing urine in the rat's bladder area was gently expelled by applying pressure after the catheter was secured. The catheter was connected to a microperfusion pump using a three-way valve equipped with a pressure transducer. Once the connections were completed, the microperfusion pump was activated. Saline was infused into the rat's bladder at a rate of 6 ml/h to simulate the urine storage process. When the bladder filled up, the rat would naturally contract its bladder to eliminate the fluid, thereby replicating the urination process. Following the operational guidelines of the urodynamic instrument system, the urodynamics test began recording once a stable graph was achieved. The software then recorded the intravesical pressure of the rat for ∼30 min. It is crucial to note that the urodynamic examination should not be repeated multiple times within a short period.

Utilizing up–down method, we gauged the bladder region's mechanical withdrawal threshold. Rats were placed in a box with a metal mesh bottom and allowed to acclimate for 30 min. Eight von Frey filaments of varying stimulation intensities (0.6, 1, 1.4, 2, 4, 6, 8, and 15 g) were selected to stimulate the skin in the bladder region through the bottom mesh. Starting with the mildest von Frey filament, we gradually intensified the stimulus until it triggered an immediate response like scratching or licking the stimulated spot. If such an immediate response was observed, the intensity was then moderated until no response could be provoked. This process was repeated until the rat's response to the stimulus stabilized.

### BDNF, TrkB, IBA-1, and IL1β expression evaluation

Rats were anesthetized with 1% pentobarbital sodium, and the L6-S1 segment of the spinal cord was isolated for examination. Protein analysis was then performed on the dorsal horn of the spinal cord using the Western blot (WB) experiment. A tissue lysis buffer was added, measured according to spinal cord weight, and the subsequent steps were performed according to the procedure outlined in the instructions. The protein concentration was determined using the micro-BCA method. A protein sample of equivalent volume (50 µg) was combined with 6 µl of loading buffer, mixed, and boiled in water for 10 min. The samples were then loaded onto an SDS–PAGE gel and underwent electrophoresis at 200 V for 45 min. The samples were then transferred onto a PVDF membrane at 100 V for 1 h. The sample was blocked at room temperature for 1 h, incubated with antibody (BDNF, 1/500, ab213323, Abcam; TrkB, 1/2,000, 13129-1, Proteintech; IBA-1: 1/1,000, ab5076, Abcam; IL-1β: 1/2,000, ab9722, Abcam) for 1 h, and then rinsed three times. It was then incubated with a horseradish peroxidase-coupled secondary antibody for 1 h, rinsed three more times, and exposed using ECL for color development for 1–3 min. Grayscale scanning was performed using ImageJ software for quantitative analysis. To accurately measure BDNF expression levels, we analyzed BDNF mRNA in the spinal dorsal horn (SDH). Tissue samples were processed for RNA extraction following the RNA Rapid Extraction Kit's protocol (ES Science, RN001). After RNA extraction, reverse transcription was conducted with Reverse Transcription Kit (ES Science, RT001). The qPCR reaction was set up with 0.4 µl of each primer (10 µmol/L), 9.2 µl of template cDNA, and 10 µl of 2xMix, totaling a volume of 20 µl. The thermal cycling conditions were 95°C for 30 s, followed by 40 cycles of 95°C for 5 s and 60°C for 30 s. Primer sequences for β-actin are forward, GGCTCCTAGCACCATGAAGA, and reverse, ACTCCTGCTTGCTGATCCAC, and, for BDNF, forward, GAGCGTGTGTGACAGTATTAG, and reverse, GTAGTTCGGCATTGCGAGTTC.

### Transcriptome sequencing analysis of SDH

Initially, the collected SDH tissues are frozen and then lysed in TRIzol reagent following the manufacturer's protocol for complete RNA extraction. Subsequently, the mRNA samples are purified and subjected to fragmentation, reverse transcription, PCR, library construction, and gene sequencing using the NovaSeq 6000 System (Illumina) provided by Lianchuan Biotechnology. The transcriptome sequencing data were analyzed utilizing a suite of bioinformatics tools, including Cutadapt, HISAT, StringTie, GffCompare, DESeq2, TBtools, Sangerbox 3.0, Cytoscape, and OmicStudio online platform (https://www.omicstudio.cn/).

### TNF-α immunofluorescence in the bladder

Rats were anesthetized by intraperitoneal injection of a 1.5% sodium pentobarbital solution, and the bladder tissue was extracted through a lower abdominal incision. The bladder was fixed in 4% paraformaldehyde and embedded in paraffin. After washing the tissue sections three times with PBS, they were blocked for 1 h with immunofluorescence blocking solution (Beyotime). The sections were then incubated overnight at 4°C with TNF-α primary antibody (1:400; 60291-1, Proteintech). Following this, the sections were incubated for 1 h in the dark with an Alexa Fluor 488-conjugated secondary antibody at room temperature. Fluorescence imaging was performed using consistent exposure and gain settings, and fluorescence intensity was visualized in 3D using the ImageJ software.

### Statistical analysis

Student's *t* test was used for statistical analysis via the GraphPad prism software.

## Results

### Significant urodynamic improvement and mechanical withdrawal threshold reduction exhibited by the EA group

**Figure 1.**
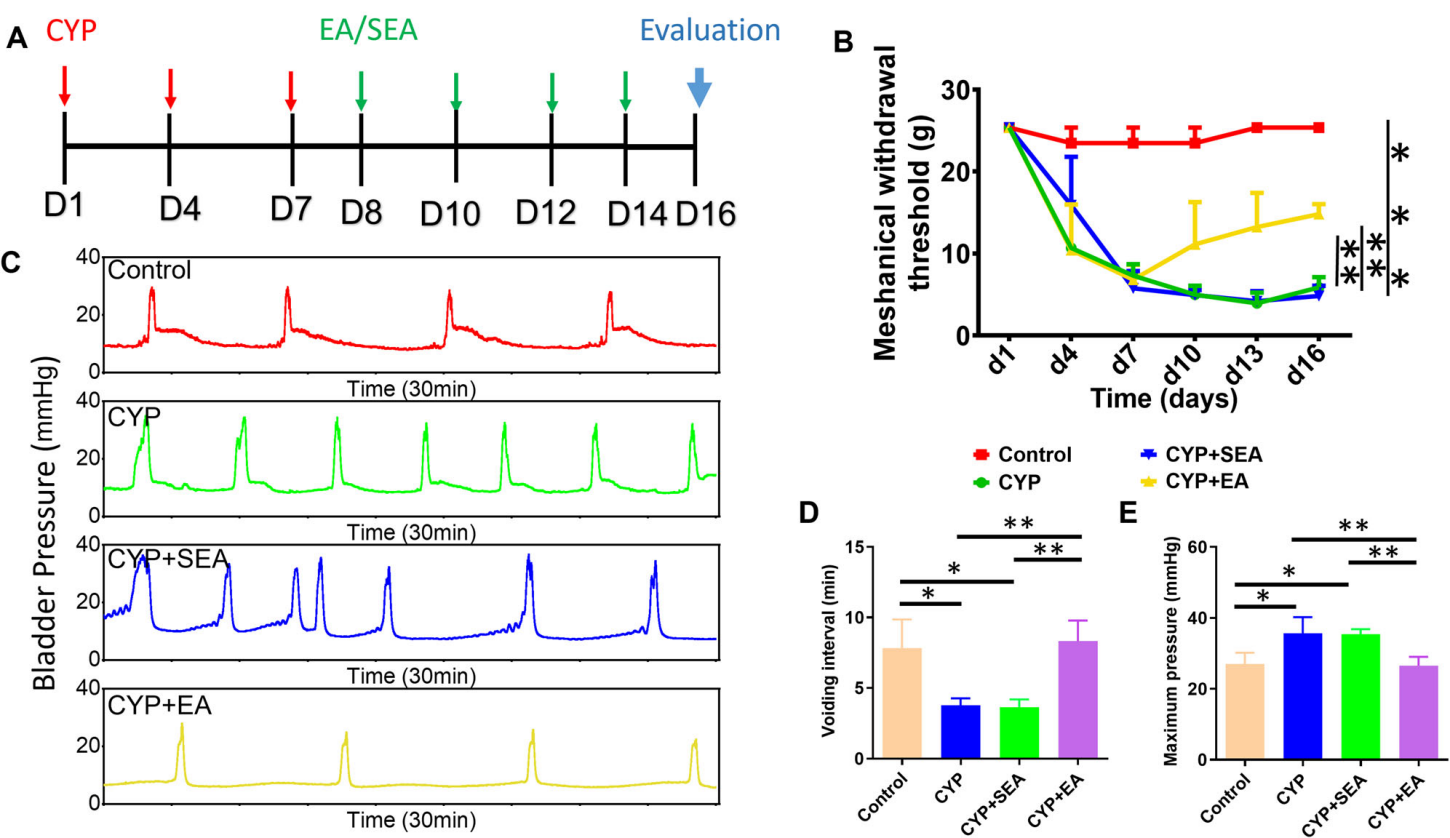
The EA group exhibited significant urodynamic improvement and a reduction in the mechanical withdrawal threshold. ***A***, Schematic of CYP injection and EA/SEA treatment. ***B***, The effect of different treatment groups on the mechanical withdrawal pain threshold in the cystitis model. ***C***, The assessment of urinary dynamics in different treatment groups. ***D***, Quantitative analysis of urination interval and (***E***) the maximum pressure in the bladder. *p < 0.05; **p < 0.01; ***p < 0.001; *n* = 4 per group for mechanical withdrawal threshold and *n* = 3 per group for urinary dynamic evaluation.

Our experiment was divided into four distinct groups: control, CYP, CYP + SEA, and CYP + EA. Figure 1*A* illustrates the establishment of the BPS model through intraperitoneal injections of CYP every 3 d, with EA treatments starting from Day 8, conducted every other day for a total of four sessions. On Day 16, urodynamic evaluations were performed on rats from different treatment groups. As depicted in Figure 1*B*, there was a marked reduction, ∼300%, in the mechanical withdrawal threshold on Day 7 in the experimental groups, compared with the control, highlighting the successful creation of the BPS model. Notably, rats treated with EA showed significant improvements in the mechanical withdrawal threshold compared with those in the SEA and BPS groups, suggesting EA's effectiveness in alleviating mechanical allodynia in BPS. Beyond pain threshold assessment, we measured bladder pressure over 30 min to evaluate the rats' urodynamic characteristics. Figure 1*C* shows a significant increase in bladder contraction frequency in the CYP-induced cystitis model, which was also present in the CYP + SEA group. Importantly, after EA treatment, bladder contractions decreased significantly, indicating improved stability of the detrusor muscle. Quantitative analyses of urination intervals and maximum bladder pressure (Fig. 1*D*,*E*) revealed substantial improvements after EA therapy, indicating that EA effectively alleviates both mechanical allodynia and bladder dysfunction in BPS.

### Downregulation of BDNF–TrkB signaling in the SDH of EA group

**Figure 2.**
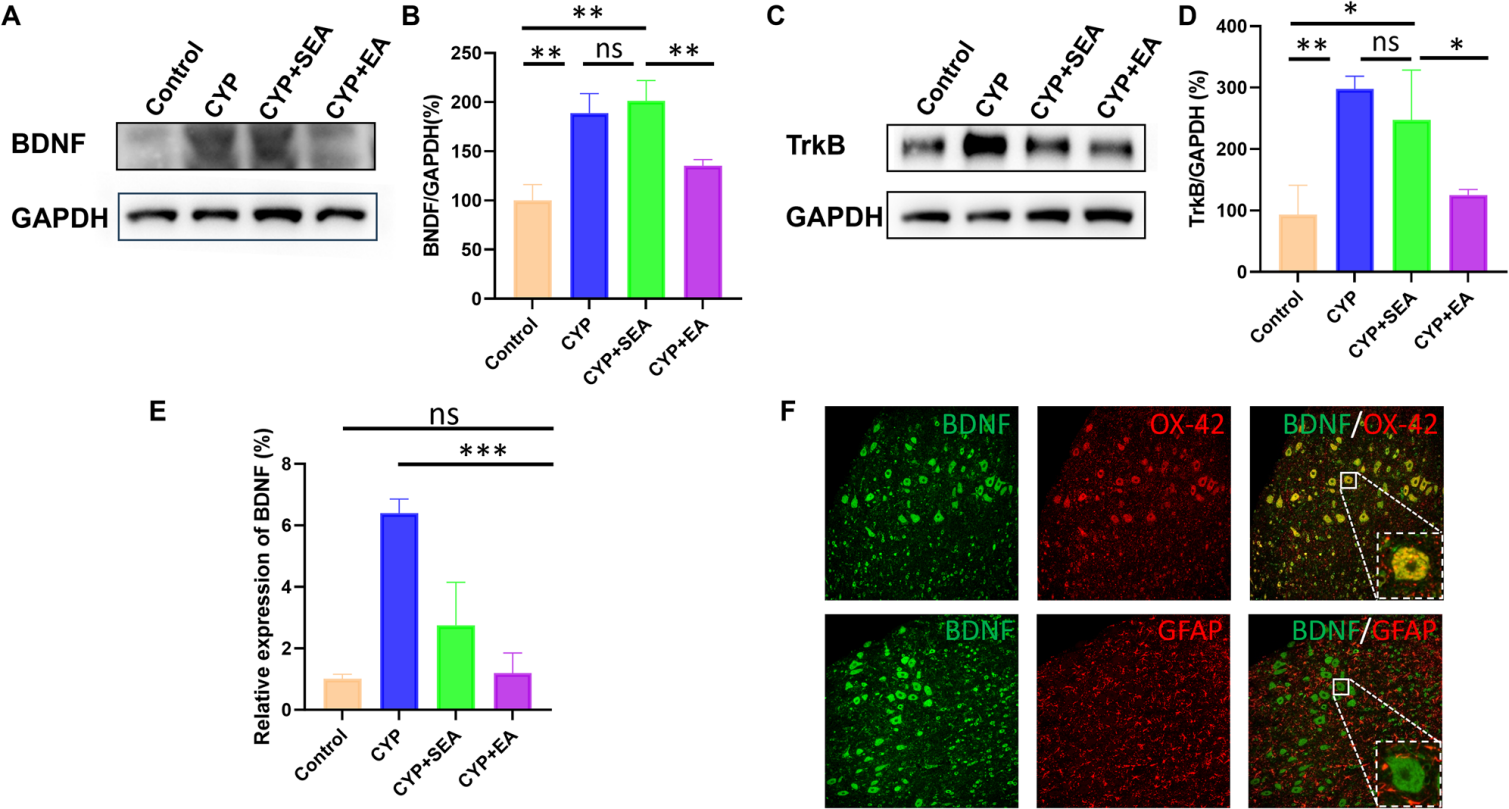
Downregulation of BDNF–TrkB signaling. ***A***, Representative BDNF expression by WB and (***B***) quantification of grayscale values. ***C***, Representative TrkB expression by WB and (***D***) quantification of grayscale values. ***E***, The mRNA expression of BDNF. ***F***, Immunofluorescence double staining assay of BDNF (green), GFAP/OX-42 (red), and colocalization (yellow) in the SDH. *p < 0.05; **p < 0.01; ***p < 0.001; *n* = 3 per group.

As shown in Figure 2*A*, an upregulation of BDNF expression was evident in the CYP-constructed cystitis model, in contrast to the control group. Meanwhile, after treating SEA, CYP rats showed no significant change in BDNF expression. Interestingly, a noteworthy reduction in BDNF expression within SDH was found following EA therapy, when compared with the CYP group. To supplement these observations, we performed a quantitative analysis of the grayscale values obtained from the WB. The results in Figure 2*B* clearly indicated that the BDNF expression in the CYP and CYP + SEA groups had almost doubled in comparison with the control group. The reduction in BDNF expression was even more pronounced with the application of EA (CYP + EA), thus reinforcing our earlier findings. In addition to BDNF, we investigated the impact of EA on the expression of the downstream protein, TrkB. As shown in Figure 2*C*, TrkB expression exhibited a significant surge in the CYP and CYP + SEA group, compared with the control group. Post-EA treatment, however, the TrkB expression was reduced, approximating the levels observed in the control group. The grayscale values of WB were again quantified to affirm these results. As evidenced in Figure 2*D*, the TrkB expression in the CYP and CYP + SEA group was 250–300% higher than the control group, but EA treatment (CYP + EA) successfully brought TrkB back to levels compared with the control group. We also analyzed the mRNA expression of BDNF in SDH. As shown in Figure 2*E*, the BDNF mRNA expression in the CYP group was six times higher than that in the control group, and following EA treatment, BDNF expression was significantly reduced. Furthermore, we conducted a fluorescent costaining analysis in the rat SDH region, focusing on the specific markers of glial cells and BDNF. Figure 2*F* illustrates that BDNF colocalized with the microglial marker OX-42 but did not colocalize with the astrocyte marker GFAP. These findings suggest that the mechanism of EA therapy might involve the inhibition of microglial cells through the BDNF–TrkB signaling pathway.

### Downregulation of the expression of IBA-1 and IL-1β in the SDH of the EA group

**Figure 3.**
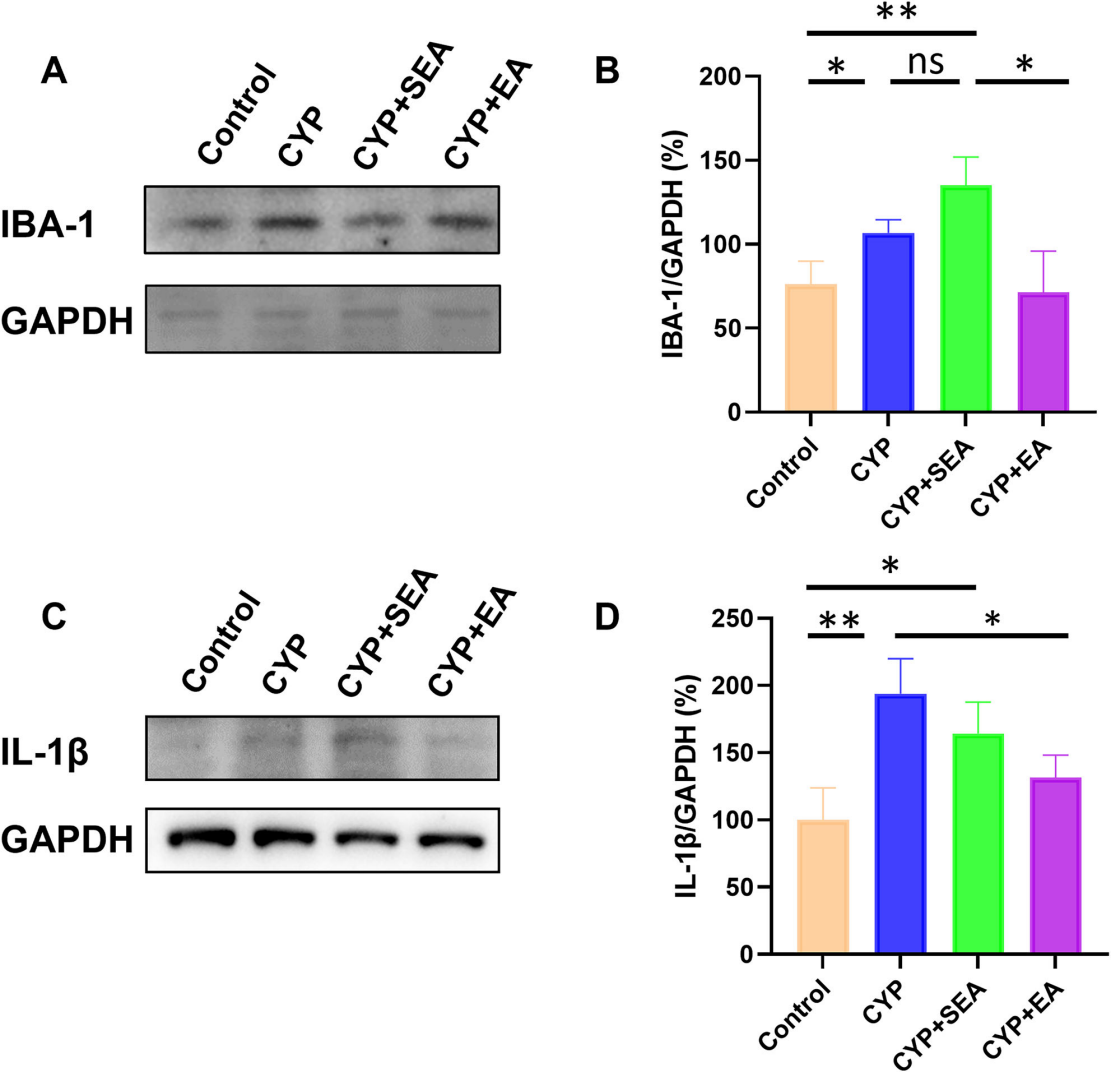
Downregulation of the expression of IBA-1(***A, B***) and IL-1β (***C, D***) in the SDH of the EA group. *p < 0.05; **p < 0.01; ***p < 0.001; *n* = 3 per group.

Upon observing the colocalization of BDNF with microglial markers, we further analyzed the microglial activation marker IBA-1 in the SDH region using WB. Figure 3*A* indicates an elevated level of microglial activation in BPS rats. Notably, this activation was reduced in the SDH region following EA therapy. In addition, the levels of the inflammatory cytokine IL-1β in the SDH region were measured, revealing a significant decrease post-EA treatment (Fig. 3*A*). These findings suggest that EA therapy may effectively inhibit microglial cell activation through the BDNF–TrkB signaling pathway.

### The administration of exogenous BDNF can negate the therapeutic effects of EA

**Figure 4.**
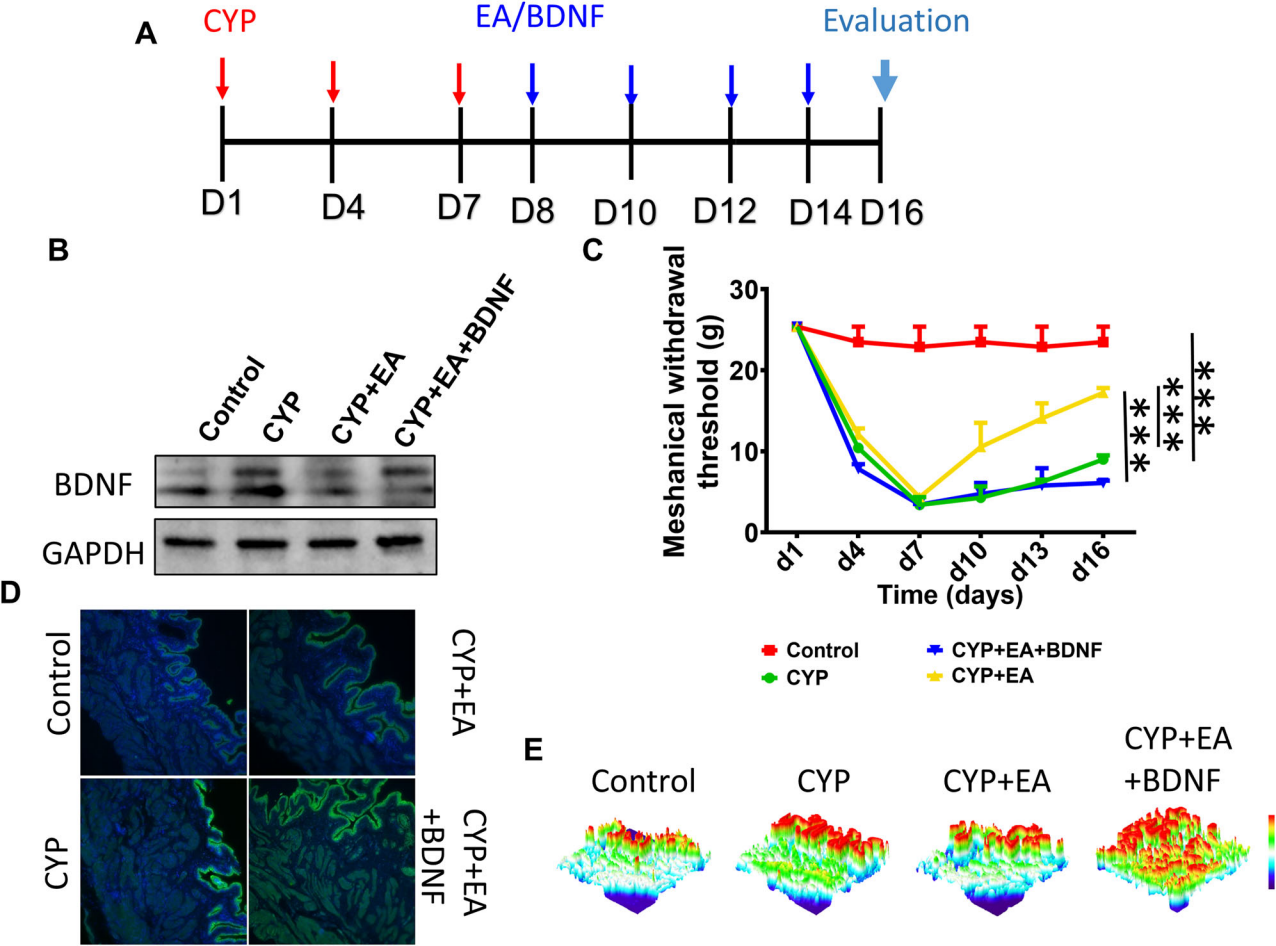
***A***, Schematic of CYP injection and EA/BDNF treatment. The administration of exogenous BDNF (***B***) can negate the therapeutic effects (***C***) of EA. Immunofluorescence of TNF-α (***D***) and fluorescence 3D visualization (***E***). *p < 0.05; **p < 0.01; ***p < 0.001; *n* = 3 per group for WB and *n* = 4 per group for mechanical withdrawal threshold evaluation.

To elucidate the pivotal role of the BDNF pathway in the efficacy of EA, we introduced exogenous BDNF to examine its potential inhibitory effects on EA's therapeutic outcomes. Figure 4*B* displays the WB analysis of BDNF across various treatment cohorts. As depicted in Figure 4*C*, EA markedly enhanced the mechanical pain threshold, indicating a significant therapeutic benefit. Intriguingly, the administration of exogenous BDNF not only nullified the benefits of EA but also resulted in a lower mechanical pain threshold when compared with the CYP control group, suggesting an exacerbation of pain sensitivity. We also assessed pathological changes in the bladder tissue by immunofluorescence. As shown in Figure 4, *D* and *E*, TNFα expression in the bladder tissue was significantly increased following CYP treatment. After EA treatment, TNFα expression decreased, while the injection of exogenous BDNF inhibited the reduction in TNFα expression. This observation underscores the critical influence of the BDNF pathway in mediating the analgesic effects of EA.

### Transcriptome sequencing analysis of SDH following EA treatment

**Figure 5.**
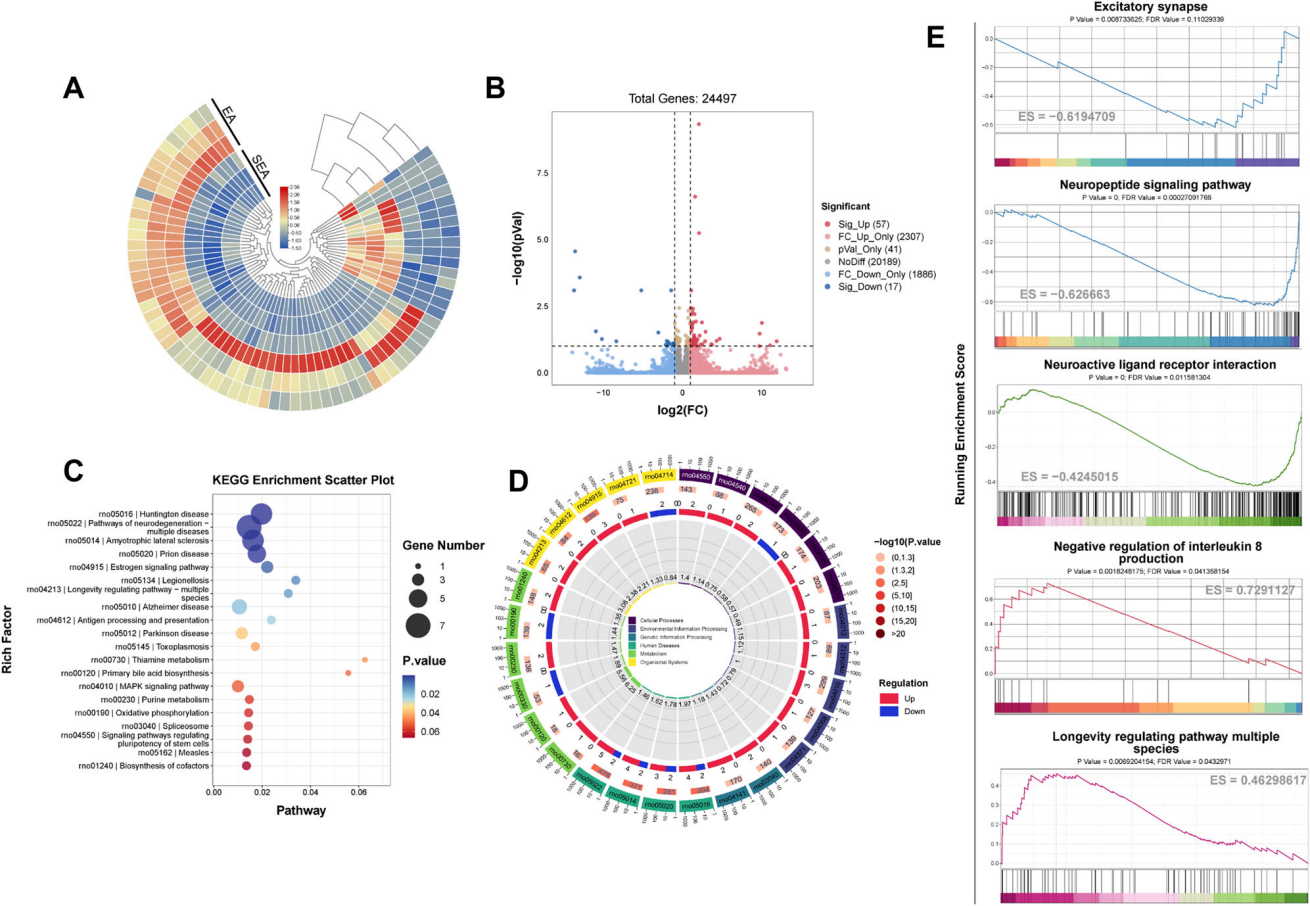
RNA sequencing analysis of SDH in rats treated with EA or SEA. ***A***, Clustering heatmap of differentially expressed genes (DEGs). ***B***, A volcano plot illustrating that DEGs are upregulated or downregulated between the two groups. ***C***, The bubble plot of KEEG enrichment analysis of DEGs, highlighting the top 20 GO terms based on the smallest *P* values. ***D***, The circos plot of the KEGG enrichment analysis. ***E***, The GSEA enrichment analysis of the transcribed gene groups. *n* = 3 for the EA and SEA group.

In an effort to delve deeper into the potential mechanisms underlying EA therapy, we conducted a transcriptome sequencing analysis of the SDH area in rats' post-EA treatment. The heatmap in Figure 5*A* illustrates the gene expression profile involved. The volcano plot in Figure 5*B* reveals that EA led to significant upregulation of 57 genes and downregulation of 17 genes. The KEGG gene database enrichment analysis, shown in Figure 5*C*, indicates improvement in gene groups associated with Huntington's disease and neurodegenerative disorders following EA. The circos plot of the KEGG enrichment analysis is shown in Figure 5*D*. The GSEA enrichment analysis of the transcribed gene groups, displayed in Figure 5*E*, indicates that EA significantly inhibited excitatory synaptic neurotransmission, neuropeptide signaling pathways, and neuroactive ligand–receptor interactions. Importantly, the treatment led to a notable upregulation of various longevity-regulating pathways.

## Discussion

EA nerve stimulation therapy, a novel treatment that deftly merges traditional Chinese acupuncture and neuroelectrical stimulation medicine, boasts a range of benefits such as extensive effects, minimal toxic side effects, economical costs, and simplicity (Li et al., 2024). Consequently, it has been widely implemented in clinical practice. Reports suggest that EA stimulation of the “Foot Sanli” and “Sanyinjiao” can partially mitigate changes in neuronal unit discharges induced by heroin addiction, suppress central sensitization, and significantly alleviate pathological pain (Xue et al., 2019). Numerous clinical and animal studies demonstrate the substantial analgesic effect of EA nerve stimulation on various forms of chronic pain, including neuralgia, inflammatory pain, persistent cancer pain, and more (Xue et al., 2019; Gong et al., 2022; Wan et al., 2023).

**Figure 6.**
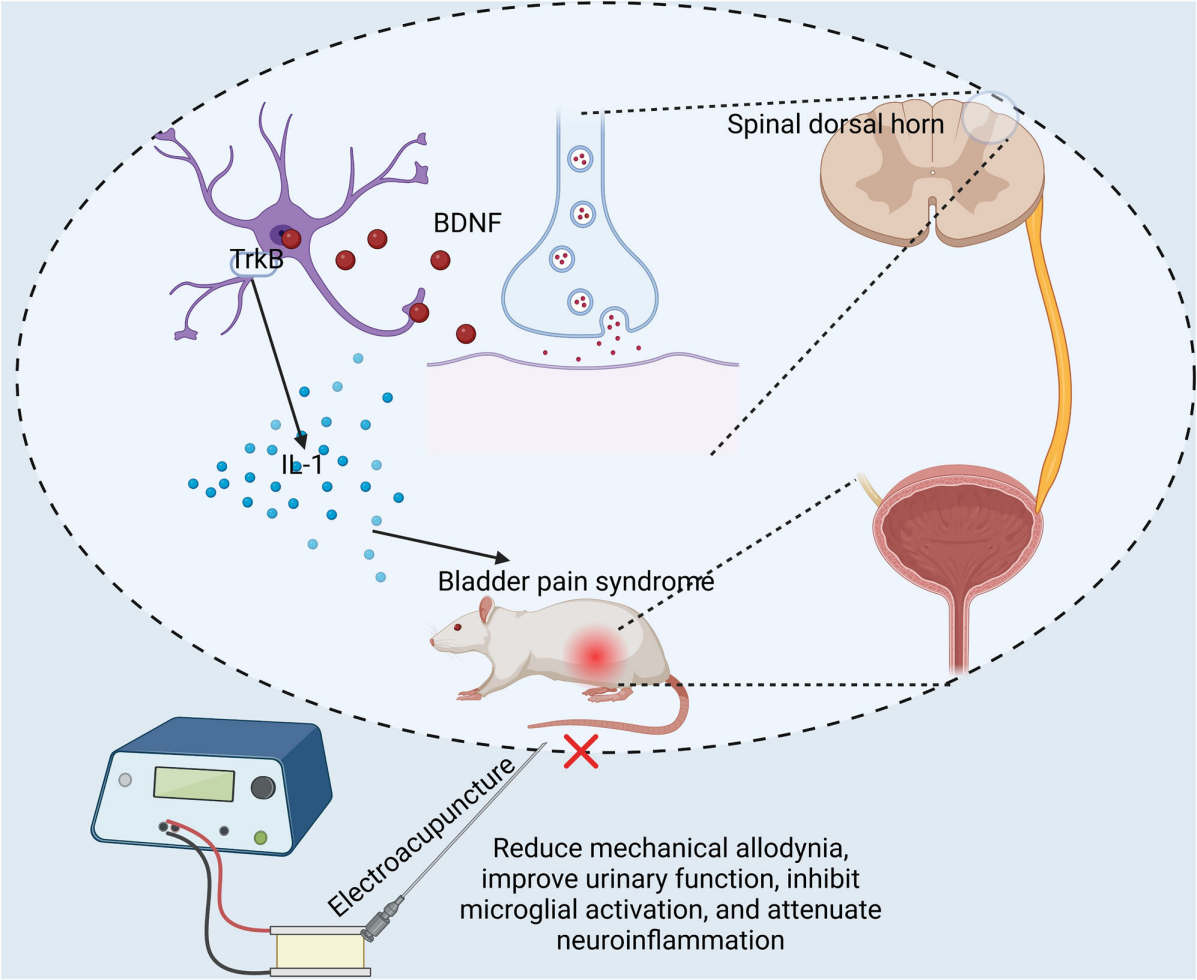
EA nerve stimulation therapy modulates BPS pain by modulating the BDNF–TrkB pathway in SDH.

It has been documented that EA stimulation can curtail the release and synthesis of substance P and calcitonin gene-related peptide at bladder afferent fiber endings, reducing the overexcitation of bladder afferent nerves, thereby playing a role in the mitigation of bladder pain (Ng et al., 2013). Moreover, some research suggests that EA may decrease the activity of spinal glial cells and astrocytic IL-1β expression in a rat model of bone cancer pain (Gong et al., 2022). Some propositions suggest that EA stimulation therapy could potentially dampen the activation of spinal glial cells (Xue et al., 2019; Gong et al., 2022). This effect might be achieved by curbing the release of pain-inducing neurotransmitters or modulators, such as excitatory amino acids and neuropeptides, from primary afferent terminals and neurons injured within the spinal cord (Gong et al., 2022). At present, the exact molecular mechanism underpinning EA's analgesic effect remains elusive. Our prior study discovered a significant increase in BDNF protein expression in the SDH area in BPS/IC animal model (Ding et al., 2020). The inhibition of its receptor could suppress glial cell and make inflammatory factors decrease, thereby reversing bladder-area pain. Based on these insights, we further investigated whether EA could potentially influence BPS/IC in this manner. As expected, our current study suggests that EA nerve stimulation therapy might alleviate BPS pain by modulating the BDNF–TrkB pathway in the SDH, inhibiting spinal glial cell activation, and reducing the release of IL-1β (Fig. 6).

### EA's significant bladder function improvement and reduction in mechanical allodynia

Research increasingly indicates that BPS/IC is linked to central sensitization driven by a neuroinflammatory response (Bresler et al., 2019; Padilla-Fernández et al., 2022). Multiple studies report that EA can effectively mitigate neuro-associate chronic pain (Xue et al., 2019; Gong et al., 2022; Wan et al., 2023; Li et al., 2024). Herein, we assessed whether EA could successfully inhibit IC. We evaluated the improvement of urodynamic properties and the mechanical withdrawal threshold brought about by EA in a BPS/IC rat model. As expected, EA treatment effectively alleviated both the urodynamic properties and mechanical withdrawal threshold in the BPS/IC model. While numerous studies are gradually unveiling the mechanisms associated with BPS/IC, effective therapies for BPS/IC are still lacking. EA treatment, being simple, effective, cost-effective, and posing low side effects, holds significant potential in treating BPS/IC.

### EA's role in downregulating BDNF–TrkB signaling in SDH

BDNF, found in both the brain and peripheral blood, plays a pivotal role in the formation of neural networks. It regulates the generation of new neurons in the adult hippocampus, enhancing the survival of neurons in the brain during development or following damage due to neurodegenerative diseases (Tuwar et al., 2024). Activation of BDNF through its TrkB receptor increases neuronal excitability, potentially leading to an increased sensitivity to pain (Andreska et al., 2023). Furthermore, BDNF activation triggers the Akt and ERK signaling pathways, influencing the apoptosis of cells (Khidr et al., 2023; Kim et al., 2024). Recent research, including our own, has identified BDNF as a crucial element in the development and progression of various types of pain, such as neuropathic, inflammatory, cancer-related, postsurgical, chronic pain, and other forms of physical discomfort (Ding et al., 2020; Jhang et al., 2023). Given that EA has been shown to effectively mitigate symptoms associated with BPS, exploring whether EA modulates these symptoms through the BDNF pathway represents a critical area of research. Our results confirm that EA regulates the BDNF–TrkB pathway in the second and third segments of the SDH. However, it is important to recognize that these segments are not only involved in bladder function regulation but also control the rectum, anus, pelvic vasculature, and so on. Whether EA's modulation of bladder function through the BDNF–TrkB pathway is influenced by other pelvic organs remains to be explored in future studies. Future clinical approaches might explore combining EA with inhibitors of specific pathways, like BDNF, to enhance therapeutic outcomes. This approach not only holds promise for improving EA's effectiveness but also establishes a theoretical basis for its broader application and promotion in managing pain.

### EA therapy inhibited the activity of microglial cells, neuroinflammation, excitatory neural conduction, and upregulated various longevity-regulating pathways

The development of neuralgia hypersensitivity is associated with the spinal cord glial cells' release of neuroactive substances, notably IL-1β, which amplifies the spinal neurons' responsiveness and increases the release of neurotransmitters like substance P and glutamate (Geng et al., 2024). This leads to heightened neural signaling at the spinal level. Microglia cells play a crucial role in supporting axonal metabolism and in the functioning of complex synaptic networks. IBA-1 is identified as a marker indicating microglial activation, which, during neuroinflammatory conditions, leads to increased synaptic engulfment and disrupted neural signaling (Chen et al., 2024). A reduction in IBA-1 and IL-1β levels in the SDH area points to a regulatory role of astrocytes in managing neuroinflammatory responses and associated pain. Looking ahead, integrating EA with strategies that target astrocyte activity or IL-1β levels may offer a synergistic approach to enhancing EA's therapeutic outcomes in treating BPS syndromes. Additionally, transcriptome analyses of the SDH region in rats subjected to EA treatment have revealed a notable weekend in neural synapse activity. Intriguingly, this treatment also resulted in significant alterations in pathways linked to Huntington's disease and other neurodegenerative disorders, highlighting EA's broad therapeutic potential. A particularly unexpected finding was the significant upregulation of pathways involved in regulating longevity, a focus area previously overlooked in EA research. These findings suggest that EA not only holds promise for neurological and pain-related conditions but may also impact the fundamental processes related to aging and longevity, opening new avenues for exploration in therapeutic applications.

### Limitations and remarkable points

This study has some limitations. We did not verify whether the activation of microglial cells is achieved through the BDNF pathway. Additionally, we did not extensively evaluate downstream inflammatory factors associated with neuralgia sensitivity. In subsequent studies, we will supplement the evaluation of related inflammatory factors such as IL-6 and P38, among others. Despite certain limitations, our study helps elucidate the involvement of the BDNF–TrkB signaling pathway in EA neurostimulation therapy for BPS/IC pain sensitivity, an insight that has not been reported hitherto. This study will offer a novel theoretical foundation for the BDNF–TrkB signaling pathway in BPS/IC, thus fostering the application and implementation of EA nerve stimulation.

## Conclusion

Our findings indicate that EA can downregulate BDNF–TrkB signaling in SDH and notably improve urodynamic properties and mechanical withdrawal thresholds. Additionally, EA can downregulate the expression of IBA-1 and IL-1β in the SDH. Moreover, EA therapy can inhibit excitatory neural conduction and upregulate various longevity-regulating pathways. In conclusion, EA nerve stimulation can effectively alleviate hyperalgesia in the BPS/IC model and shows therapeutic potential in other diseases. This effect may be achieved by downregulating the BDNF–TrkB signaling pathway, thus inhibiting the activation of astrocytes in the SDH and reducing the release of inflammatory cytokines.

## Synthesis

Reviewing Editor: Jennifer Dulin, Texas A&M University Decisions are customarily a result of the Reviewing Editor and the peer reviewers coming together and discussing their recommendations until a consensus is reached. When revisions are invited, a fact-based synthesis statement explaining their decision and outlining what is needed to prepare a revision will be listed below. The following reviewer(s) agreed to reveal their identity: Agnieszka Balkowiec.

The manuscript is much improved, but some concerns remain. First, the visual abstract is not sufficiently detailed to convey the main conclusion of the study. It helps to understand the methodology, but not the results and conclusions. The authors focus on the accurate visual representation of individual objects rather than the mechanistic explanation of the observed phenomena. Second, some issues regarding the selectivity of the results need to be addressed. Please see below.

Reviewer comments:

The authors have adequately addressed many of my concerns. Thank you for that. Unfortunately, my major concern regarding the selectivity of the observed phenomena (Q.2) has not been addressed at all.

1) SDH samples show massive upregulation of BDNF mRNA and protein, most likely not limited to the neuronal populations serving the bladder. My suggestion to examine BDNF upregulation in specific subpopulations of spinal neurons (e.g. at the mRNA level by in situ hybridization; colocalization with neuronal tracer from the bladder, etc.) has not been addressed.

2) There is no evidence that the inflammatory process is restricted to the bladder. The authors focus on the evidence for the inflammation within the bladder, but my concern is that the inflammatory process is widespread (after the intraperitoneal injection of cyclophosphamide) and not limited to the bladder.

Again, there is no evidence for the selectivity of either, BDNF upregulation in the subpopulation of SDH neurons specifically supplying the bladder, and inflammation limited to the bladder. Without such evidence, it is difficult to validate the finding that the highly selective, largely limited to the innervation of the bladder, EA nerve stimulation results in a widespread downregulation of BDNF in many SDH neuronal populations serving tissues far beyond the bladder.

At a minimum, this concern should be addressed in the Discussion section.

## Author Response

Reply to the comments of Reviewer Q1: The manuscript is much improved, but some concerns remain. First, the visual abstract is not sufficiently detailed to convey the main conclusion of the study. It helps to understand the methodology, but not the results and conclusions. The authors focus on the accurate visual representation of individual objects rather than the mechanistic explanation of the observed phenomena. Second, some issues regarding the selectivity of the results need to be addressed. Please see below.

R1：Thank you for your suggestion. We agree that the visual abstract was not sufficiently detailed to effectively convey the main conclusions of the study. Therefore, we have revised the visual abstract (Figure 6) to better illustrate the mechanistic explanation of the observed phenomena. Regarding your second concern, we will address it in the subsequent sections.

Fig.6. EA nerve stimulation therapy modulates BPS pain by modulating the BDNF-TrKB pathway in SDH Q2: SDH samples show massive upregulation of BDNF mRNA and protein, most likely not limited to the neuronal populations serving the bladder. My suggestion to examine BDNF upregulation in specific subpopulations of spinal neurons (e.g. at the mRNA level by in situ hybridization; colocalization with neuronal tracer from the bladder, etc.) has not been addressed.

R2：Thank you for your careful review and valuable comments on our manuscript. Your feedback has deepened our understanding of the importance of BDNF upregulation in specific spinal nerve subpopulations, which is an extremely important research direction. Regarding your suggestion to further investigate the expression of BDNF upregulation in spinal nerve subpopulations by in situ hybridization technology or colocalization with neuronal tracer from the bladder, we understand that this would provide more detailed and compelling evidence for our study. However, due to resource limitations, neuro tracing presents a challenge for us, we were unable to include this experiment in the current version of the manuscript. We sincerely acknowledge that the absence of this experiment may limit the comprehensiveness of our research. We have incorporated this experiment into our future research plans and will make every effort to supplement and improve it in our future work.

We have added "Our results confirm that EA regulates the BDNF-TrkB pathway in the 2nd and 3rd segments of the SDH. However, it is important to recognize that these segments are not only involved in bladder function regulation but also control the rectum, anus, pelvic vasculature, and so on. Whether EA's modulation of bladder function through the BDNF-TrkB pathway is influenced by other pelvic organs remains to be explored in future studies." in the Discussion section. The revised section is highlighted in yellow. Once again, thank you for your guidance and assistance in our research. Your suggestions have undoubtedly provided direction for our future research. We will continue to strive to improve the quality of our research and look forward to the opportunity to report our progress to you.

Q3：There is no evidence that the inflammatory process is restricted to the bladder. The authors focus on the evidence for the inflammation within the bladder, but my concern is that the inflammatory process is widespread (after the intraperitoneal injection of cyclophosphamide) and not limited to the bladder.

Again, there is no evidence for the selectivity of either, BDNF upregulation in the subpopulation of SDH neurons specifically supplying the bladder, and inflammation limited to the bladder. Without such evidence, it is difficult to validate the finding that the highly selective, largely limited to the innervation of the bladder, EA nerve stimulation results in a widespread downregulation of BDNF in many SDH neuronal populations serving tissues far beyond the bladder.

R3：Thank you for your suggestion. We agree with your point. After intraperitoneal injection, CYP is metabolized into acrolein, a highly reactive aldehyde, which is then excreted by the kidneys into the bladder (Chang, Yu et al. 2024). As acrolein accumulates in the bladder, it interacts with the umbrella cells of the urothelium, thereby inducing an inflammatory response (Tay and Grundy 2023). While intraperitoneal injection of CYP is bladder-specific, a single high dose of CYP (150 mg/kg) can also lead to inflammation in other organs (Augé, Gamé et al. 2020). To minimize the impact on other organs, the dosage was optimized to 75 mg/kg, administered every three days for a total of three doses, ensuring that the inflammatory response remains localized to the bladder without significant infiltration, consistent with the dosing regimen used in our study(Yuridullah, Corrow et al. 2006, Augé, Gamé et al. 2020).To further confirm whether intraperitoneal cyclophosphamide injection causes widespread effects beyond the bladder, we performed histological staining of the heart, liver, spleen, and lungs of rats after CYP-induced modeling. As shown in Fig.S1, no significant histological differences were observed in these organs between the CYP-treated rats and the control group.

Fig.S1. Hematoxylin and eosin (H&E) staining of the tissue sections from the heart, liver, spleen, and lung of the rats.

References:

Augé, C., X. Gamé, N. Vergnolle, P. Lluel and S. Chabot (2020). "Characterization and Validation of a Chronic Model of Cyclophosphamide-Induced Interstitial Cystitis/Bladder Pain Syndrome in Rats." Front Pharmacol 11: 1305.

Chang, Y. C., C. Y. Yu, C. Dong, S. L. Chen and W. W. Sung (2024). "Divergent histopathological and molecular patterns in chemically induced interstitial cystitis/bladder pain syndrome rat models." Sci Rep 14(1): 16134.

Tay, C. and L. Grundy (2023). "Animal models of interstitial cystitis/bladder pain syndrome." Front Physiol 14: 1232017.

Yuridullah, R., K. A. Corrow, S. E. Malley and M. A. Vizzard (2006). "Expression of fractalkine and fractalkine receptor in urinary bladder after cyclophosphamide (CYP)-induced cystitis." Auton Neurosci 126-127: 380-389.
